# Genetic evidence for a worldwide chaotic dispersion pattern of the arbovirus vector, *Aedes albopictus*

**DOI:** 10.1371/journal.pntd.0005332

**Published:** 2017-01-30

**Authors:** Mosè Manni, Carmela R. Guglielmino, Francesca Scolari, Anubis Vega-Rúa, Anna-Bella Failloux, Pradya Somboon, Antonella Lisa, Grazia Savini, Mariangela Bonizzoni, Ludvik M. Gomulski, Anna R. Malacrida, Giuliano Gasperi

**Affiliations:** 1 Department of Biology and Biotechnology, University of Pavia, Pavia, Italy; 2 Laboratory of Medical Entomology, Environment and Health Unit, Morne Jolivière, Institut Pasteur of Guadeloupe, Les Abymes, Guadeloupe (French West Indies); 3 Department of Virology, Arboviruses and Insect Vectors Unit, Institut Pasteur, Paris, France; 4 Department of Parasitology, Chiang Mai University, Chiang Mai, Thailand; 5 Computational Biology Unit, Institute of Molecular Genetics-National Research Council, Pavia, Italy; North Carolina State University, UNITED STATES

## Abstract

**Background:**

Invasive species represent a global concern for their rapid spread and the possibility of infectious disease transmission. This is the case of the global invader *Aedes albopictus*, the Asian tiger mosquito. This species is a vector of medically important arboviruses, notably chikungunya (CHIKV), dengue (DENV) and Zika (ZIKV). The reconstruction of the complex colonization pattern of this mosquito has great potential for mitigating its spread and, consequently, disease risks.

**Methodology/Principal findings:**

Classical population genetics analyses and Approximate Bayesian Computation (ABC) approaches were combined to disentangle the demographic history of *Aedes albopictus* populations from representative countries in the Southeast Asian native range and in the recent and more recently colonized areas. In Southeast Asia, the low differentiation and the high co-ancestry values identified among China, Thailand and Japan indicate that, in the native range, these populations maintain high genetic connectivity, revealing their ancestral common origin. China appears to be the oldest population. Outside Southeast Asia, the invasion process in La Réunion, America and the Mediterranean Basin is primarily supported by a chaotic propagule distribution, which cooperates in maintaining a relatively high genetic diversity within the adventive populations.

**Conclusions/Significance:**

From our data, it appears that independent and also trans-continental introductions of *Ae*. *albopictus* may have facilitated the rapid establishment of adventive populations through admixture of unrelated genomes. As a consequence, a great amount of intra-population variability has been detected, and it is likely that this variability may extend to the genetic mechanisms controlling vector competence. Thus, in the context of the invasion process of this mosquito, it is possible that both population ancestry and admixture contribute to create the conditions for the efficient transmission of arboviruses and for outbreak establishment.

## Author Summary

*Aedes albopictus*, the Asian tiger mosquito is the most invasive species in the world. Over the past 30 years, from its native tropical Southeast Asia it rapidly spread across the world, and it is currently present in several tropical, subtropical and temperate countries due to its high ecological plasticity. Its dramatic global expansion has resulted in public health concerns as a consequence of its vector competence for several arboviruses, such as chikungunya, dengue and Zika, which are the most prevalent pathogens of humans. The identification of the migration routes and, consequently, the demographic origin of mosquito adventive populations is a priority to prevent local outbreaks of viral infection. Using a combined population genetics and Bayesian approach, we found that the worldwide invasion of this mosquito has not occurred through a step-by-step expansion process from the native Southeast Asia. A recent and chaotic human-mediated propagule distribution allowed adventive populations to be established in the Indian Ocean, Mediterranean Basin, and North America regions. We have detected high intra-population variability, which may co-operate with the genetic mechanisms controlling vector competence.

## Introduction

The term ‘successful biological invasion’ includes the detrimental impact on human health, economy and native biodiversity [1–3]. Indeed, invasive species represent a global concern especially because of their rapid spread and competitive nature, and the possibility of transmission of infectious diseases. The ability of a species to establish and become invasive can be influenced by a variety of factors including its life history traits, ecological and environmental constraints, and the intensity of propagule pressure [4, 5]. The on-going intensification of the global transportation networks offers new opportunities for species to disperse, even into remote regions [6, 7]. A new term, Anthropocene, has been suggested [8] to name the present epoch characterized by human impacts on biota. By creating new bioconnectivity among ecosystems, human activity is creating new ecological niches of adaptation for invasive species and this represents an increasingly important factor in contemporary evolution [9].

*Aedes* mosquito species [10] offer a variety of case histories of successful invasions, mainly human mediated [11], and provide examples of how human activities have impacted their evolutionary histories [7, 12, 13]. This is the case for important arbovirus vector species such as *Ae*. *aegypti*, *Ae*. *albopictus* and *Ae*. *japonicus japonicus* [14]. Their eggs, in favourable conditions of humidity and temperature, can survive long transportation times so that a large number of viable eggs can be accidently transported [15–17]. On the other hand, anthropogenic activities also create new breeding and trophic niches of adaptation in close proximity to human living sites, impacting their relationships with humans [12, 13]. The global invasion of *Ae*. *albopictus* (Skuse, 1894), the Asian tiger mosquito, is an example of human-aided transport [18] with important public health implications. This mosquito is listed among the top hundred most dangerous invasive species [19]. Originally, it was a zoophilic forest species which, from tropical East Asia [20, 21], spread first to the Indian and Pacific Ocean islands [22] and, during the 1980s, rapidly expanded its range, also across temperate regions, in Europe, the Americas and Africa [14, 23–26]. Ecological, demographic, behavioural genetic/genomic studies indicated that this mosquito is able to tolerate climate/environment interactions that differ from its native range [27–29]. One of the most important adaptive life history traits is its ability to diapause in the presence of unfavorable conditions [30–32]. The rapid global spread of *Ae*. *albopictus* is creating public concern due to its vector competence for at least 20 arboviruses, notably chikungunya (CHIKV), dengue (DENV) and Zika (ZIKV) viruses [16, 33–42]. Indeed, outside its original range this mosquito has been increasingly involved in local autochthonous transmission of chikungunya and dengue in many places where it has become established, including La Réunion, continental Europe, Africa, the Americas and Japan [43, 44]. In Europe, the first chikungunya outbreak took place in 2007 in Italy in the Ravenna/Cesena area, with more than 200 confirmed cases [45, 46]. Later outbreaks occurred in France [47] and Croatia [48]. Therefore, the identification of important dispersal routes has great potential for mitigating the spread of the mosquito and for preventing/reducing the risk of outbreaks [49]. Plausible routes of introduction of *Ae*. *albopictus* at the global scale have been inferred from historical and observational data [50]. These data, however, are not informative for the demographic histories of the adventive populations in the different countries. Population genetic approaches using mtDNA, rDNA and microsatellite markers are providing, at different spatial scales, data on the phylogeographical relationships among populations and on their genetic diversity [51–58]. However, these data are highly disparate for providing a synthesis of the demographic history of this mosquito [59].

Previously, we suggested that the global expansion of *Ae*. *albopictus* was primarily supported by a chaotic propagule distribution mediated by human activity [60]. In order to support this hypothesis, here we combine classical population genetics analyses and Approximate Bayesian Computation (ABC) approaches to infer the demographic history of populations from representative countries in the Southeast Asian native range and in the more recently colonized areas.

Specifically, we provide information on the origins of populations from Southeast Asia (*i*.*e*., China, Thailand, Japan), the Indian Ocean, the Mediterranean Basin, the Pacific Ocean islands and North America. Since tiger mosquito populations display differences in vectorial capacity for different arboviruses [35, 38, 61–63], the outcomes from this paper are valuable to develop models that may predict the risk of vector outbreaks [59].

## Methods

### Mosquito collections

During a period from 2010 to 2012, tiger mosquito samples were collected as eggs from public land in ten different localities: three from the native Asian region (China, Japan, Thailand) and seven from invaded areas: Indian Ocean (La Réunion Island), Mediterranean Basin (Italy, Albania, Greece), Pacific Ocean (Hawaii) and North America (U.S.A.) (Table 1). These localities were chosen as sampling sites because data on the history and ecology of their well-established populations are available. To avoid any seasonality effect on population size and dynamics, especially in temperate regions, eggs were collected in the peak of population size (July-August), *i*.*e*., not in the diapause or pre-diapause period [32]. A standardized sampling protocol was adopted in order to minimize the inbreeding effect [59, 64]. In each locality, 15 to 17 ovitraps were placed at a distance of least 500 m one to another, and at least 40 eggs/ovitrap were obtained. The taxonomic identification of the emerging adults was confirmed using morphological keys [65]. Three individuals emerging from each ovitrap collection were pooled for each locality, and part was supplied to our laboratory as ethanol-preserved specimens, from which total genomic DNA was individually extracted.

**Table 1:** *Aedes albopictus* populations sampled in areas in which the presence of this mosquito has been historically recognised. Dates refer to the first observational record in the country of the sampling sites.

Area	Country	First historical record	Sample site	Sample name	Sample size	Latitude	Longitude	Date of collection
East Asia	Japan	-	Nagasaki	JP	13	32°75’N	129°88’E	2011
	China	-	Xiamen	CN	10	24°47’N	118°09’E	2011
	Thailand	-	Ban Rai	TH	30	15°30’N	99°45’E	2010
Indian Ocean	Réunion	18^th^ Century [37]	Saint Pierre	RE	30	21°32’S	55°47’E	2010
Mediterranean	Greece	2003 [66, 67]	Athens	GR	29	37°98’N	23°73’E	2011
basin	Albania	1979 [37]	Tirana	AL	24	41°33’N	19°83’E	2011
	Italy	1990 [37]	Cesena	IT1	31	44°14’N	12°25’E	2010
			Brescia	IT2	26	45°54’N	10°22’E	2010
Pacific Ocean	Hawaii	~1900 [68]	Oahu	HI	29	21°43’N	158°00’W	2012
North America	U.S.A.	1985 [69]	Manassas (Virginia)	VA	30	38°75’N	77°47’W	2012

### Microsatellite genotyping

Each mosquito was screened for seventeen previously characterized microsatellite loci [60], *i*.*e*., from Aealbmic1 to Aealbmic17. These loci, which are spread across the genome, ensure sufficient power to detect genetic variability even in relatively small samples [70]. PCR reactions and fragment resolution were performed as we previously described [60]. The TANDEM program [71] for the automated binning of allele lengths (with additional manual checking) was used to overcome the problems of genotyping errors. In cases in which microsatellite amplification was not successful or scoring was uncertain, re-extraction of DNA was performed.

### Data analyses

Genetic variation within each locality was estimated in terms of average numbers of alleles (*n*_a_), number of private alleles (*n*_p_) and frequency of private alleles (*A*_p_) using GenAlEx 6.5 [72, 73]. The average number of alleles and private alleles were also computed at the individual level: n_a_/n and n_p_/n, respectively. The inbreeding index (*F*_IS_) was obtained using FSTAT V.2.9.3.2 [74]. Observed and expected heterozygosity and Pairwise-*F*_ST_ [75] values were computed using Microsatellite Analyser V.4.05 [76]. The statistical significance of each *F*_ST_ value was assessed by comparison of the observed value with the values obtained in 10,000 matrix permutations and Bonferroni corrections were applied. Linkage disequilibrium between pairs of loci in each sample (100 batches, 1000 iterations per batch) and deviations from Hardy-Weinberg equilibrium (HWE) at each locus/sample combination were examined with GENEPOP V. 4.2 [77, 78] and the statistical significance was assessed following Bonferroni corrections [79]. The allelic polymorphic information content and null allele frequencies (*A*_n_) for each locus were estimated using CERVUS [80].

### Population structure and demographic inference

The relationships among populations were assessed using Principal Coordinate Analysis in GenAlEx V.6.4 [73]. Bayesian clustering analysis in STRUCTURE V 2.3.2 [81–83] was used to infer population structure using the admixture model and assuming independent allele frequencies. The burn-in was set to 500,000 steps and was followed by 1,000,000 Markov Chain Monte Carlo replications. All runs were repeated 20 times for each number of possible clusters (*K*), set between 1 and 10 (*i*.*e*., the number of samples). The appropriate number of genetic clusters was determined by plotting the log probability (L(*K*)) and ΔK across multiple runs [84] as implemented in STRUCTURE HARVESTER [85]. Finally, the programs CLUMPP [86] and DISTRUCT [87] were used to average replicate runs and to generate bar graphs of structure results, respectively.

### Definition of invasion scenarios

To infer the invasion routes followed by *Ae*. *albopictus*, competing hypotheses regarding population divergence at the global scale were compared using an Approximate Bayesian Computation (ABC) method, as implemented in DIYABC v2.0 [88]. Because of the huge number of possible scenarios (10!) that could be tested with the ten considered populations, a step by step approach has been adopted considering subgroups of populations, starting from the putative ancestral area to new invaded regions. Southeast Asia was assumed as the ancestral region considering that it has been suggested to be the species home range. On this basis, plausible invasion scenarios were set considering the historical knowledge of the first record in invaded countries and the data obtained from population genetic analyses (*i*.*e*., population structure and ancestry). The competing scenarios were set using prior definitions and distribution of demographic parameters, as described in S1 Table. We took into account the effective population size, the timing in which split or admixure events occurred, the number of founders which contributed to the establishment of adventive populations, the duration of the eventual bottlenecks occuring during colonization and the rate of admixture, if considered. The prior parameters were kept deliberately broad when no prior information were available. This is the case for the effective population sizes, for which broad priors [500–100,000] with uniform distributions were chosen for all the populations. The time range of each event was kept less broad when it was supported by historical data. The timing of events was expressed in numbers of generations back in time. Since the number of generations/year is dependent on bioclimatic conditions, we assumed around 4–7 generations/year in temperate regions, and 12–17 generations in tropical regions.

Although *Ae*. *albopictus* has great potential for rapid population growth [89], uncertainty regarding the duration of the bottleneck was taken into account. Therefore, we assumed a bottleneck period with an uniform prior distribution bounded between 0 and 50 generations. During colonization, the number of founder individuals for each colonization event was described as *NF* and its size was drawn from a uniform distribution bounded between 1 and 100 individuals. If necessary, the rate of admixture between two populations was drawn in a uniform distribution and set from 0.001 to 0.999. For the genetic parameters, the microsatellite Generalized Stepwise Mutation model was considered, and a mean microsatellite mutation rate across loci was set between 10^−5^ and 10^−3^. The parameter of geometric distribution was specified with a uniform prior distribution bounded between 0.1–0.3. For each locus the above considered mutation parameters were allowed to vary using Gamma distributions. Finally, the possibility of single nucleotide insertion/deletion mutations were considered with a mean frequency of 10^−8^–10^−5^ (S1 Table).

The genetic variation within and among the populations was summarized using sets of statistics conventionally used in ABC analyses: the mean number of alleles per locus, the mean expected heterozygosity, the mean allelic size variance, the overall pairwise F_ST_ values, and the Garza-Williamson index (the number of alleles in a population divided by the range in allele size)[90]. This index helps to discriminate populations which have experienced recent losses in genetic variability from those that have been stable for a long time. One million simulated datasets were generated for each scenario. Competing scenarios were compared by calculating their posterior probabilities using a polychotomous logistic regression on the 1% of simulated data closest to the observed data [91]. Confidence in scenario choice was evaluated by computing type I and type II errors [92]. Once the most likely scenario was identified for each analysis, the posterior distributions of genetic and demographic parameters were estimated. This was achieved by computing a local linear regression on the 1% of the simulated data closest to our observed dataset, after applying a logit transformation to the parameters value. The goodness-of-fit of the estimation procedure was also evaluated by performing a model checking computation by generating 1000 pseudo-observed data sets with parameter values drawn from the posterior distribution given the most likely scenario.

## Results

### Intra-specific variation

The considered seventeen microsatellite loci, scored in 252 mosquitoes across the 10 localities from Asia to America, displayed different levels of polymorphism in terms of number of alleles, ranging from 2 alleles (Aealbmic17) to 24 alleles (Aealbmic5). The number of alleles per locus in each population is provided in S2 Table and the original genotype data are available in Open Science Framework data repository (doi: 10.17605/OSF.IO/U4JRP). Overall these loci display a mean Polymorphic Information Content of 0.54 suggesting that they are sufficiently informative for assessing the degree of population variability and structuring of this mosquito. Results of Fisher’s exact tests, after sequential Bonferroni correction [79], revealed significant departures from HWE in 13 out of 170 population/locus combinations. However, the HWE departures were not concentrated at any locus or in any population. No consistent pattern of linkage disequilibrium between any particular pair of loci was observed, therefore the 17 loci were considered as independent and their variability might well reflect genome-wide patterns across populations.

Variability at these loci appeared relatively high across the ten populations (Table 2) but in the Asian region, the China (CN), Japan (JP), and Thailand (TH) populations displayed a relatively higher level of variation and this was particularly evident when the number of alleles is referred to the number of individuals (n_a_/n). While private alleles were detected in all populations, the highest proportion was estimated in the Asian region and in La Réunion island. An estimate of variability distribution in and among the populations (AMOVA) indicated that most variation (85%) occurred within individuals, whereas only about 15% of total variation was detected among populations.

**Table 2:** Genetic variability estimates of ten *Ae*. *albopictus* samples from different geographical regions. n_a_, mean number of alleles; n_a_/n mean number of alleles/individual; n_p_, number of private alleles; n_p_/n mean number of private alleles/individual; *A*_p_, mean frequency of private alleles; *H*_O_, mean observed heterozygosity; *H*_E_, mean expected heterozygosity; *A*_n_, mean frequency of null alleles.

Populations	n_a_	n_a_/n	n_p_	n_p_/n	A_p_	H_O_	H_E_	A_n_
JP	4.71	0.36	5	0.38	0.06	0.41	0.55	0.09
CN	3.71	0.37	4	0.40	0.09	0.41	0.56	0.10
TH	6.06	0.20	8	0.27	0.03	0.44	0.57	0.08
RE	5.65	0.19	13	0.43	0.04	0.53	0.58	0.03
GR	4.29	0.15	4	0.14	0.02	0.42	0.50	0.05
AL	4.18	0.17	5	0.21	0.04	0.38	0.48	0.07
IT1	4.94	0.16	2	0.06	0.02	0.47	0.51	0.03
IT2	3.65	0.14	4	0.15	0.04	0.42	0.51	0.06
HI	3.65	0.12	1	0.03	0.02	0.34	0.45	0.08
VA	4.88	0.16	4	0.13	0.03	0.45	0.52	0.05

JP, Japan; CN, China; TH, Thailand; RE, La Réunion; GR, Greece; AL, Albania; IT1, Italy1/Cesena; IT2, Italy2/Brescia; HI, Hawaii; VA, Virginia (U.S.A.).

### Population structure

**Fig 1:**
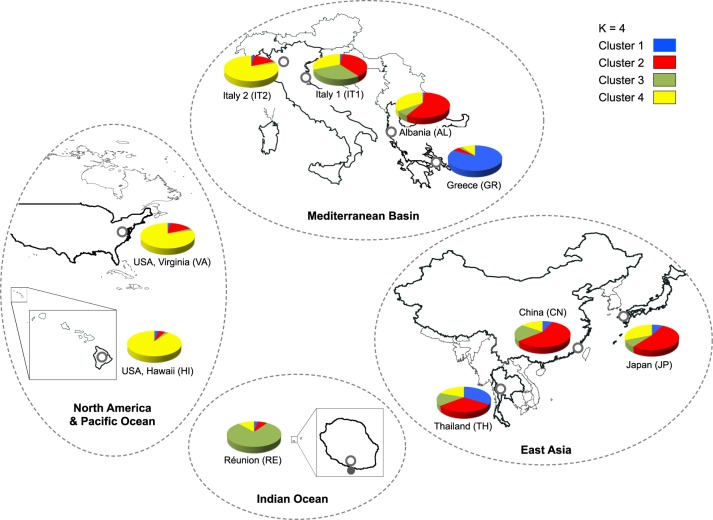
Geographical representation of the co-ancestry distribution of *Ae*. *albopictus* mosquitoes. A total of 252 individuals from 10 populations were sampled from the supposed East Asian native area, and from newly colonized regions. The four colours represent the four hypothetical clusters as defined by STRUCTURE. The figure was based on freely available maps [93], and modified using Adobe Illustrator CC 2014 (Adobe Systems Inc., San Jose, CA, USA).

Pairwise F_ST_ values were significantly different from zero in all the comparisons (Table 3). The lowest estimates were detected among the Asian populations (0.028 between CN-JP, 0.051 between TH-JP). By contrast, within the Mediterranean area, there was heterogeneity in terms of pairwise-*F*_ST_ values. Two geographically close regions such as Greece and Albania were the most differentiated (F_ST_ = 0.187), but Greece displayed a certain genetic affinity with Thailand (F_ST_ = 0.083), and Albania with China (F_ST_ = 0.075). In America, Virginia shared greatest genetic affinity with the Asian populations (especially with Japan) but the lowest F_ST_ value was with the IT2 (Brescia) Italian population (F_ST_ = 0.057). La Réunion, besides being linked to the Asian region, was genetically related to the Mediterranean area, with the exclusion of Greece (F_ST_ = 0.148). The overall absence of correlation between genetic differentiation (F_ST_) and geographical distance was confirmed by the R^2^ value = 0.009, *P* = 0.649 (S1 Fig). These population relationships were evident from the Principal Coordinate Analysis plot (S2 Fig). Also the Bayesian method implemented in STRUCTURE showed that the coancestry proportion among individuals of the ten populations was not consistent with their geographic proximity. The natural logarithm of the likelihood of the data, ln(P(X/K)), increased from *K* = 1 to *K* = 4 and then gradually reached a plateau towards 10, suggesting that the genetic clustering of the ten populations was 4. The individual mosquitoes from the ten populations were then assigned to each of four clusters with a certain probability value (Fig 1; S3 Table). The individuals of China were mainly represented in cluster 2 (red) together with Japan and partially with Thailand. Cluster 2 could be therefore identified as the Asian cluster. The co-ancestry of the Mediterranean populations was heterogeneous: Albania was tightly related (57%) to the Asian cluster 2, while Greece was mainly represented in cluster 1 (blue)(83%) related only to Thailand (34%). Also the ancestry of the two North Italian populations (IT1, IT2) was heterogeneous. IT2 (Brescia) shared about 80% of coancestry in cluster 4 (yellow), with the Hawaiian and North American (Virginia) samples. By contrast, the ancestry of IT1 (Cesena) was fragmented between Asian cluster 2 (38%), American cluster 4 (31%), but also with La Réunion cluster 3 (green)(29%).

**Table 3:** Matrix of geographic distance (km) (above diagonal) and pairwise-F_ST_ values (below diagonal) among wild *Ae*. *albopictu*s samples.

	JP	CN	TH	RE	GR	AL	IT1	IT2	HI	VA
JP		1,473	3,631	9,931	9,068	9,117	9,400	9,414	7,127	11,682
CN	0.028*		2,199	8,478	8,731	8,894	9,314	9,379	8,464	12,925
TH	0.051	0.047		6,301	7,739	8,044	8,621	8,752	10,648	14,164
RE	0.079	0.073	0.068		7,391	7,886	8,514	8,737	16,575	15,295
GR	0.132	0.147	0.083	0.148		500	1,180	1,399	8,339	8,474
AL	0.081	0.075	0.099	0.079	0.187		431	563	8,107	7,985
IT1	0.051	0.055	0.068	0.060	0.143	0.080		139	7,873	7,294
IT2	0.078	0.108	0.068	0.077	0.119	0.123	0.058		7,759	7,079
HI	0.108	0.114	0.117	0.115	0.143	0.110	0.097	0.101		7,677
VA	0.066	0.097	0.083	0.084	0.130	0.100	0.093	0.057	0.106	

JP, Japan; CN, China; TH, Thailand; RE, La Réunion; GR, Greece; AL, Albania; IT1, Italy1/Cesena; IT2, Italy2/Brescia; HI, Hawaii; VA, Virginia (U.S.A).

* F_ST_ value which resulted not significantly different from zero at *p*>0.05 (ns) after Bonferroni correction.

### Approximate Bayesian Computation (ABC method) to infer colonisation histories

**Fig 2:**
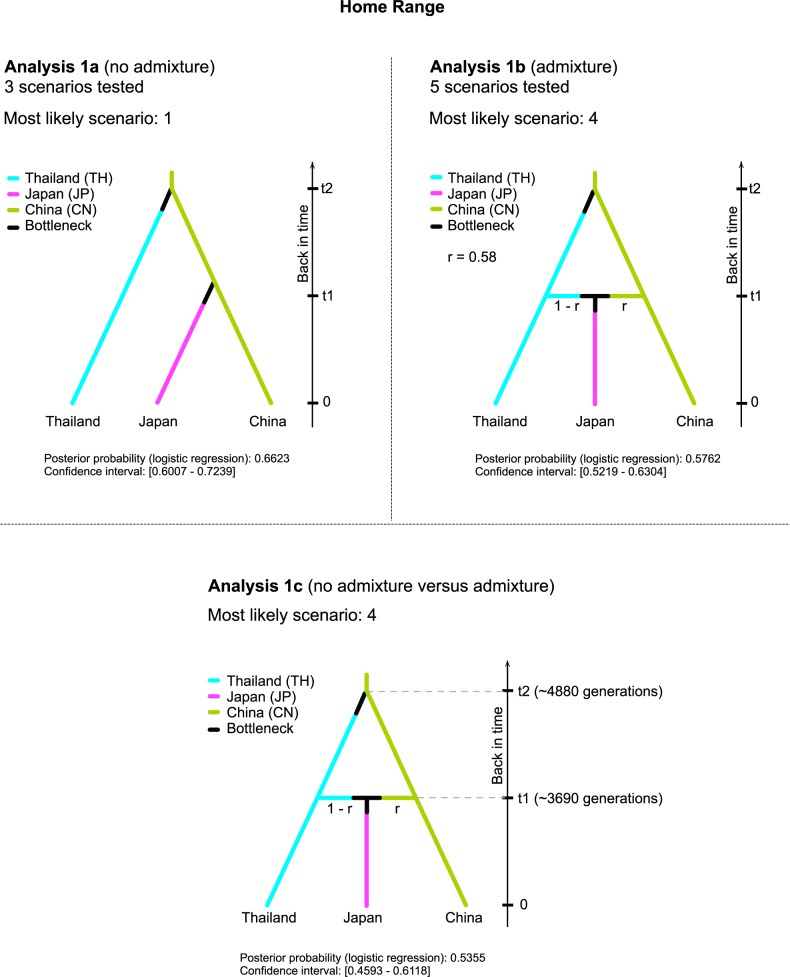
Graphical representation of the most likely scenario of each set of scenarios describing the dynamics of samples within the native area using ABC methods.

**Fig 3:**
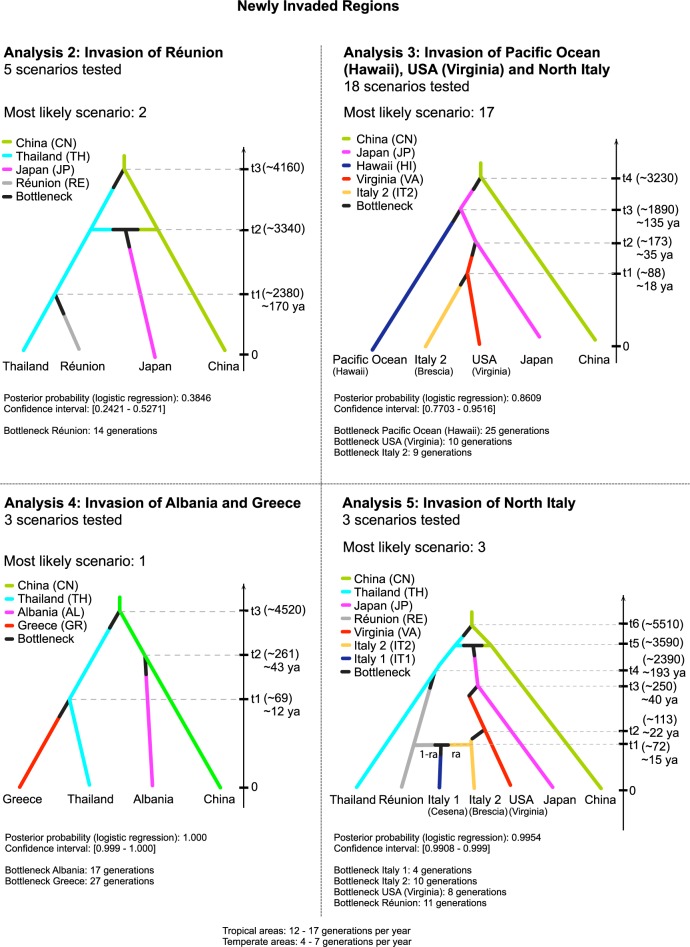
Graphical representation of the most likely scenario of each set of scenarios describing the dynamics of samples in newly invaded areas using ABC methods.

Five separate sequential ABC analyses (Table 4) were performed to disentangle the steps in the colonization history: from the expansion in the putative Asian home range (analyses 1a-b-c) to the invasion of La Réunion Island (analysis 2), North America (analysis 3) and Mediterranean region (analyses 4–5). The graphical representation of the most-likely scenario obtained for each analysis, along with the parameters inferred from genetic data, are presented in Figs 2 and 3. Analysis 1, which consisted of three sequential steps (1a-b-c), considered the relationships among the Asian samples collected in China, Thailand and Japan (Table 4, Fig 2). In analysis 1a, the divergence among these populations has been assumed in the absence of admixture events. Here, the highest posterior probability was for scenario 1 (P = 0.66) in which the first step of population divergence occurred between China and Thailand, and a second split between China and Japan. When admixture events were considered (analysis 1b), the highest posterior probability supported scenario 4 (P = 0.58), in which Japan arose as an admixture between China and Thailand. Both scenarios indicated China as an ancestral population. However, we cannot unambiguously demonstrate that the derivation of Japan might include an admixture event between China and Thailand. Indeed, the comparison (analysis 1c) between the scenarios with and without admixture resulted in a slightly higher posterior probability (P = 0.54) for the admixture hypothesis (scenario 4), which, however, had no strong statistical support. Indeed the 95% Confidence Intervals (CI) of the two scenarios (P_S4_CI = [0.46, 0.61]; P_S1_CI = [0.39, 0.54]) were partially overlapping.

**Table 4:** Posterior probabilities and 95% confidence intervals for the competing invasion scenarios of *Ae*. *albopictus* within native and newly colonised regions, as estimated by the ABC approach.

Region	Analysis		Scenario	Posterior Probability	Confidence interval
**Home Range (East Asia)**					
	1a	No Admixture	**1 CN→TH; CN→JP**	**0.6623**	**[0.6007,0.7239]**
			2 JP→CN; JP→TH	0.2703	[0.1422,0.3984]
			3 TH→CN; TH→JP	0.0675	[0.0000,0.1826]
	1b	Admixture	**4 CN→TH; CN+TH→JP**	**0.5762**	**[0.5219,0.6304]**
			5 CN→JP; CN+JP→TH	0.2522	[0.2045,0.2998]
			6 TH→JP; TH+JP→CN	0.0454	[0.0283,0.0625]
			7 JP→TH; JP+TH→CN	0.0613	[0.0428,0.0799]
			8 JP→CN; JP+CN→TH	0.0649	[0.0484,0.0815]
	1c	No Admixture vs Admixture	1 CN→TH; CN→JP	0.4645	[0.3882,0.5407]
			**4 CN→TH; CN+TH→JP**	**0.5355**	**[0.4593,0.6118]**
**Newly Invaded Regions**					
**La Réunion**	2		1 CN→TH; CN+TH→JP; CN→RE	0.2477	[0.1002,0.3953]
			**2 CN→TH; CN+TH→JP; TH→RE**	**0.3846**	**[0.2421,0.5271]**
			3 CN→TH; CN+TH→JP; TH+JP→RE	0.2800	[0.1602,0.3998]
			4 CN→TH; CN+TH→JP; JP+CN→RE	0.0253	[0.0000,0.1210]
			5 CN→TH; CN+TH→JP; JP→RE	0.0624	[0.0000,0.1598]
**Hawaii, North America, North Italy**	3		1 CN→JP; CN→HI; CN→VA; CN→IT2	0.0008	[0.0000,0.0112]
			2 CN→JP; CN→HI; JP→VA; CN→IT2	0.0003	[0.0000,0.0107]
			3 CN→JP; CN→HI; CN→VA; JP→IT2	0.0000	[0.0000,0.0105]
			4 CN→JP; JP→HI; CN→VA; JP→IT2	0.0000	[0.0000,0.0105]
			5 CN→JP; CN→HI; HI→VA; CN→IT2	0.0000	[0.0000,0.0105]
			6 CN→JP; CN→HI; CN→VA; HI→IT2	0.0001	[0.0000,0.0106]
			7 CN→JP; CN→HI; CN→VA; VA→IT2	0.0177	[0.0000,0.0394]
			8 CN→JP; JP→HI; HI→VA; CN→IT2	0.0000	[0.0000,0.0105]
			9 CN→JP; CN→HI; JP→VA; VA→IT2	0.1069	[0.0197,0.1940]
			10 CN→JP; CN→HI; HI→VA; VA→IT2	0.0017	[0.0000,0.0121]
			11 CN→JP; JP→HI; JP→VA; CN→IT2	0.0000	[0.0000,0.0105]
			12 CN→JP; CN→HI; HI→VA; HI→IT2	0.0000	[0.0000,0.0105]
			13 CN→JP; JP→HI; HI→VA; VA→IT2	0.0055	[0.0000,0.0168]
			14 CN→JP; CN→HI; HI→VA; JP→IT2	0.0000	[0.0000,0.0105]
			15 CN→JP; JP→HI; CN→VA; VA→IT2	0.0008	[0.0000,0.0112]
			16 CN→JP; CN→HI; JP→VA; HI→IT2	0.0005	[0.0000,0.0109]
			**17 CN→JP; JP→HI; JP→VA; VA→IT2**	**0.8609**	**[0.7703,0.9516]**
			18 CN→JP; JP→HI; JP→VA; HI→IT2	0.0047	[0.0000,0.0167]
**Albania, Greece**	4		**1 CN→TH; CN→AL; TH→GR**	**1.0000**	**[0.9999,1.0000]**
			2 CN→TH; CN→AL; AL→GR	0.0000	[0.0000,0.0000]
			3 CN→TH; CN→AL; CN→GR	0.0000	[0.0000,0.0000]
**North Italy**	5		1 CN→TH; CN+TH→JP; TH→RE; JP→VA; VA→IT2; RE→IT1	0.0034	[0.0000,0.0077]
			2 CN→TH; CN+TH→JP; TH→RE; JP→VA; VA→IT2; IT2→IT1	0.0012	[0.0000,0.0029]
			**3 CN→TH; CN+TH→JP; TH→RE; JP→VA; VA→IT2; IT2+RE→IT1**	**0.9954**	**[0.9908,0.9999]**

JP, Japan; CN, China; TH, Thailand; RE, La Réunion; GR, Greece; AL, Albania; IT1, Italy1/Cesena; IT2, Italy2/Brescia; HI, Hawaii; VA, Virginia (U.S.A).

In the subsequent four scenarios the origins of the adventive populations were considered.

Analysis 2 focused on the invasion of La Réunion Island. To determine which Asiatic population mostly contributed to the invasion of La Réunion, 5 alternative hypotheses were simulated (Table 4). Scenario 2, where La Réunion mosquitoes were derived from Thailand, presented the highest posterior probability (P = 0.3846, 95% CI [0.2421, 0.5271]) (Table 4; Fig 3). However, note that the 95% CI for this hypothesis overlapped with those of La Réunion's derivation from China and/or from an admixture between Thailand and Japan.

Analysis 3 focused on the colonization history of Hawaii, North America and North Italy (IT2-Brescia). A total of 18 scenarios were designed to test different hypotheses of introduction. The results unambiguously indicated scenario 17 (Table 4; Fig 3), in which the Hawaiian and North American (Virginia) mosquitoes were derived from Japan. North America acted as a ‘bridgehead’[94] from which the mosquitoes were introduced into North Italy. The choice of this scenario was highly supported by a posterior probability (P = 0.8609, 95% CI [0.7703, 0.9516]) never overlapping with those of the other competing scenarios.

In analysis 4, the relationships among the nearby Mediterranean populations of Albania and Greece were considered together with their putative source, China and Thailand respectively, as suggested by the STRUCTURE data. The introduction of mosquitoes from China to Albania and from Thailand to Greece was supported by the highest posterior probability (P = 1.0000, 95% CI [0.9999, 1.0000]) never overlapping those of the other competing scenarios (Table 4; Fig 3).

Finally, we tried to disentangle the relationship between the two North Italian samples, IT1-Cesena and IT2-Brescia. Out of the three considered scenarios (Table 4), that with the most support (P = 0.9954, 95% CI [0.9908, 0.9999]) suggested a different origin for the two Italian samples: IT1 was the result of an admixture between La Réunion and individuals from IT2 (Table 4; Fig 3). The La Réunion source area shows a much higher contribution to the genetic pool of the IT1 (Cesena) sample, as the admixture rate was estimated to be 60%.

Interestingly, the computations of the bottleneck severity parameter from posterior distributions provided overall support for minimal to moderate bottleneck severity during the invasions with the duration of the bottlenecks varing from 4 (IT1, Cesena) to 27 generations (GR, Greece).

## Discussion

**Fig 4:**
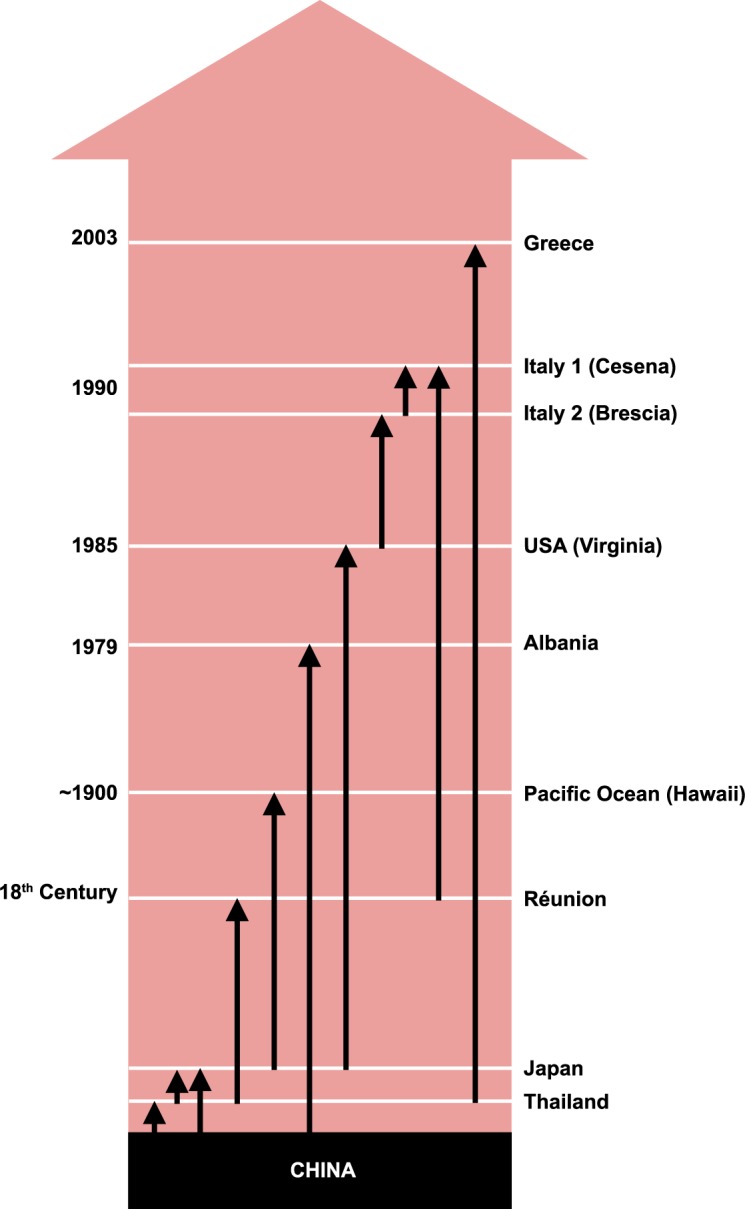
Demographic history of Asian, Indian Ocean, American and Mediterranean Basin populations as deduced by genetic and historical data.

Our results clearly confirm that: 1) Southeast Asia, and especially China, is the native range of *Ae*. *albopictus*, 2) the invasion process is supported by a chaotic propagule distribution, which may play an important role in maintaining the genetic diversity and in the establishment of the adventive populations, and 3) the complex invasion pathways inferred from genetic data are congruent with the historical documentation concerning the presence of this species. All these outcomes are summarized in Fig 4.

### Genetic status of *Aedes albopictus* within East Asia

It has been suggested that, before the global invasion, the presence of climatically suitable areas across East Asia enabled rapid population growth and expansion allowing the persistence of this mosquito in widely interconnected populations from Southeast China to the Indochinese Peninsula [21, 54]. Recent changes in the environment created by urbanization allowed the species to become very abundant in rural and suburban/urban areas across East Asia [16, 95, 96]. Within these areas we sampled three high density populations, one from Southeast China (Fujian province), one from Thailand (Lampang province) and one from South Japan (Nagasaki area) to investigate their ancestral relationships and their actual genetic status. Based on the analysis of the genetic variability, it appears clear that the samples from China, Thailand and Japan represent populations displaying genetic features expected in large populations in a native area, such as a high number of alleles, coupled with a high number of private alleles occurring at high frequencies [97]. This observation is especially interesting for the Chinese sample, which was collected in the Southeastern coastal region, one of the native areas of this mosquito [21]. The low levels of differentiation and the high co-ancestry values identified among China and the other two Asian samples allows us to confirm that, in the native range of expansion, these populations maintain high genetic connectivity, revealing their ancestral common origin. The conclusive ABC analysis indicates that, among the East Asian populations, China appears to be the oldest population. Japan, in turn, has a high probability to represent a population emerging from an admixture between the more ancient populations from China and Thailand. A certain level of admixture may be caused by multiple introductions during the initial stages of invasion, which, in turn, may cause an increase in genetic diversity favouring expansion and adaptation [98, 99]. The historical networks of trade and exchanges across East Asian maritime space, the ‘China seas’ [100], may have facilitated the diffusion of propagules. It is noteworthy that our Japanese sample was collected in the Southwestern region, which represents an ancestral area in which the mosquito was well established for a long time, and from where it expanded its infestation range northwards [101, 102].

### Invasion processes out of East Asia

Outside East Asia, our data depict a picture of multiple migration patterns, with some erosion of variability. Historically, since the 18^th^ century this mosquito is believed to have spread, along with humans, from Southeast Asia to Madagascar and the Indian Ocean islands [103]. As a consequence, in this region the mosquito had a long history of colonization and it has had time to adapt and probably to undergo differentiative processes [55]. Our La Réunion sample has the genetic signature of these processes: it maintains ancestry with the East Asian populations, but also displays the signs of differentiative processes. It is highly polymorphic with a great number of private and rare alleles, all features typical of an old and well established population.

After its spread to islands in the Indian Ocean, the reported invasion of this species to new areas started around 1900, when *Ae*. *albopictus* began a chaotic global expansion that continues to the present [104]. In the New World, the species was introduced into the Hawaiian islands at the end of the 18^th^ century, probably from Japan [105, 106] and it became the primary mosquito species [107]. In continental USA, the first detection was in Texas in the 1980s, imported from Japan/Asia as a consequence of the complex ramifications of international business and the trade of tyres [68]. The mosquito spread to several USA states up the East Coast and North-Eastern region due to internal trade and multiple introductions from Japan/South Asia and Hawaii [108]. These historical records parallel our STRUCTURE and ABC data in corroborating the hypothesis that Japan, among the Asian samples, represented an important source area from which the mosquito was introduced into the USA.

In the Mediterranean region the outcomes of cluster analysis and scenario-testing portray a complex invasion pattern with most of the established populations being admixtures resulting from independent introductions scattered across time. The genetic discontinuity between the populations from geographically related countries, such as Albania, Greece and Italy, is a clear example. The Albanian population displays high co-ancestry with China from where it was introduced in 1979, when China was one of Albania’s few trading partners [50, 109]. The Albanian infestation was the first recorded invasion outside the Oriental and Australasian regions and the first in the Mediterranean area. Although the mosquito became established in Albania, there were no reports in any other European country until 1990, when it was detected in Italy [110, 111], and only in 2003 in Greece [66]. The evolutionary scenarios modelled in ABC indicate the Greek population from the Athens area as an independent and recent derivation from Thailand. How this region could have been implicated in the introduction of this mosquito is an open question, although it is noteworthy that the Athens Piraeus Port is the hub for the international shipping trade from Thailand to the Mediterranean region [112]. Regarding the presence of *Ae*. *albopictus* in North Italy, our data clearly show that, although North America acted as an important ‘bridgehead’ [94], different and unrelated introductions contributed to the establishment of populations in North Italy. Indeed, while one of the two Italian samples, Brescia (IT2), shares high ancestry with the Americas, the other geographically close sample, Cesena (IT1), appears to be the result of admixture events mainly with La Réunion. Cesena is an area of tourism in Emilia Romagna on the West coast of the Adriatic sea, with a port, which is on the cargo shipping route from La Réunion/Mauritius to Italy [113]. We cannot exclude the hypothesis that the introduction of La Réunion mosquitoes into the Cesena area may be correlated with this on-going shipping network.

### Global invasion of *Ae*. *albopictus* as a vector of arboviruses: what consequences for disease outbreaks?

It is likely that independent and also trans-continental introductions of *Ae*. *albopictus* may have facilitated the rapid establishment of adventive populations through admixture of unrelated genomes. As a consequence, a great amount of intra-population variability has been detected, and it is likely that this variability may extend to the genetic mechanisms controlling vector competence [114]. Intriguingly, competence for CHIKV, DENV and ZIKV has been found to be highly variable within and among populations [35, 38, 61, 63, 115]. Successful viral transmission is indeed strictly dependent on specific combinations of mosquito genome and viral genetic characteristics [61, 62]. Thus, in the context of the invasion process of this mosquito, it is possible that both population ancestry and admixture may contribute to create the conditions for the efficient transmission of arboviruses and for outbreak establishment. An example is the 2007 chikungunya outbreak in North Italy (the first in Europe), in an area comprising Cesena/Ravenna [45, 116]. This outbreak was caused by *Ae*. *albopictus* autochthonous transmission of the CHIKV strain carrying the A226V mutation [45, 117] previously identified during the large 2005–2006 outbreak in La Réunion island [118, 119]. Thus, these North Italian mosquitoes share similar high competence for the same CHIKV genotype with the La Réunion mosquitoes, with which, as we have shown, they have high co-ancestry.

These observations stimulate the need to explore how the dynamics of different mosquito genetic backgrounds, during the invasion processes, may have impacted variation in competence within and among the adventive populations. Moreover, we must consider that the spread of this mosquito occurred across different environmental conditions. Thus, the variation in competence between the eco-geographic populations may be the result of interactions between unexplored genetic factors and the complex environmental landscapes [25, 120]. Knowledge of these interactions is crucial in the assessment of the risk of arbovirus outbreaks in old and newly invaded areas where *Ae*. *albopictus* has become established.

## Supporting Information

S1 Fig Relationship between pairwise F_ST_-values and the geographical distances for the 10 *Ae*. *albopictus* populations. (DOC)

S2 Fig Three-dimensional plot of principal coordinate analysis based on similarity matrix derived from *Ae*. *albopictus* microsatellite data. (TIF)

S1 Table Definition and prior distribution of parameters used in the ABC analyses for describing the set of scenarios investigated for the reconstruction of the invasion of *Ae*. *albopictus*. (DOC)

S2 Table Number of alleles per population at each microsatellite locus. (DOCX)

S3 Table Average coefficient of ancestry obtained from a STRUCTURE run with *K* = 4 for 252 individuals of *Ae*. *albopictus* from 10 samples collected in different eco-geographical areas. (DOC)
