# Supplementary figures and images for: Genetic evidence for a worldwide chaotic dispersion pattern of the arbovirus vector, Aedes albopictus
Source: PLoS Negl Trop Dis. 2017 Jan 30;11(1):e0005332. doi: 10.1371/journal.pntd.0005332 (PMC5300280; doi:10.1371/journal.pntd.0005332)

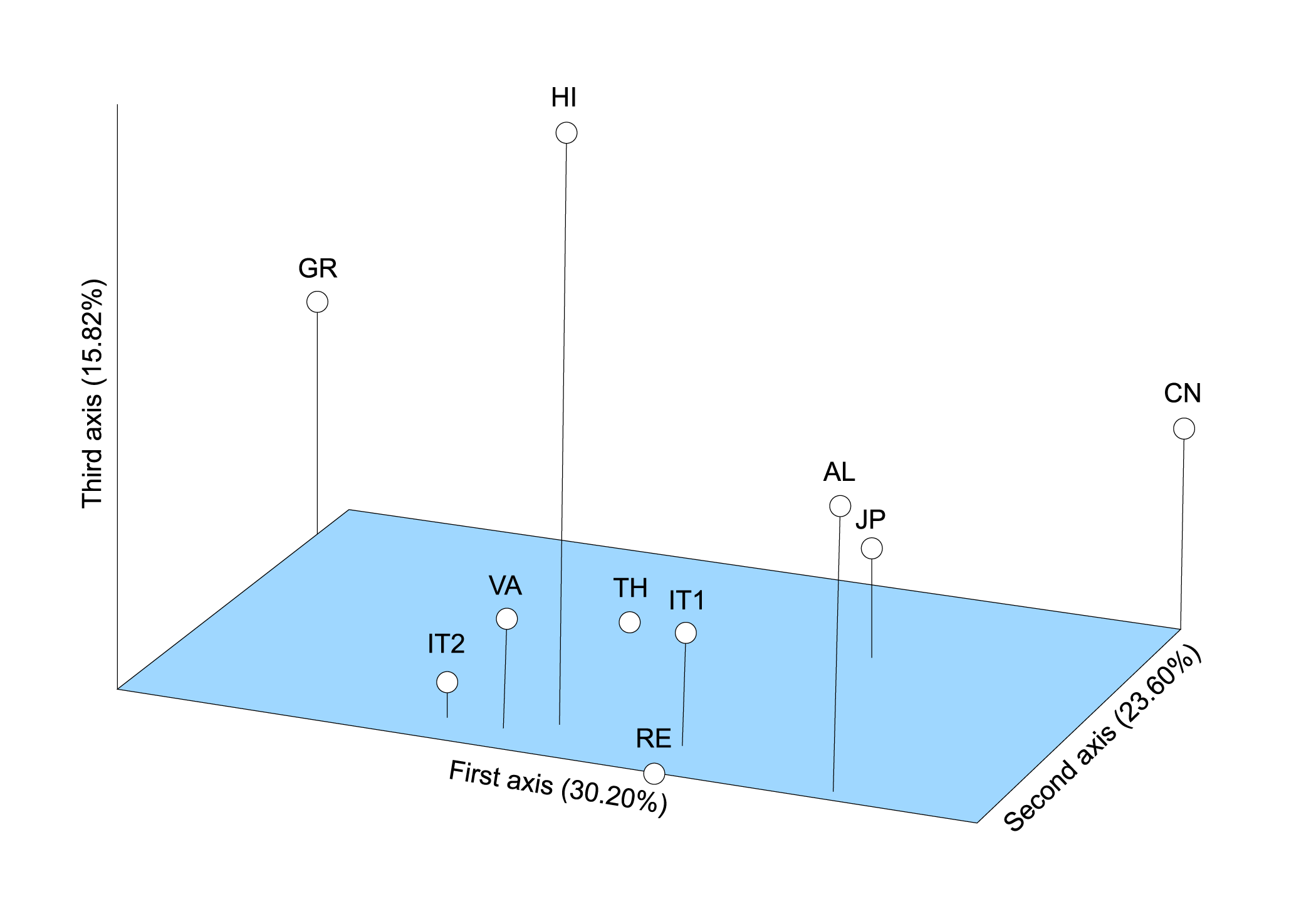

Supplement: S2 Fig — (TIF) [file pntd.0005332.s002.tif]
