# Supplementary material for: Genetic evidence for a worldwide chaotic dispersion pattern of the arbovirus vector, Aedes albopictus
Source: PLoS Negl Trop Dis. 2017 Jan 30;11(1):e0005332. doi: 10.1371/journal.pntd.0005332 (PMC5300280; doi:10.1371/journal.pntd.0005332)
Supplement: S1 Table — (DOC) [file pntd.0005332.s003.doc]

**S1 Table.** Definition and prior distribution of parameters used in the ABC analyses for describing the set of scenarios investigated for the reconstruction of the invasion of *Ae. albopictus*.

| Parameter | Distribution | Interval |
| --- | --- | --- |
| Effective population size, *N_i_* | Uniform | 500 - 100000 |
| Number of founders, *NF_i_* | Uniform | 1 - 100 |
| Duration of bottleneck, *Db_i_* | Log-Uniform | 0 - 50 |
| Founder event of Italian populations (IT2, IT1) | Uniform | 20 - 140 |
| Founder event of VA population | Uniform | 120 - 220 |
| Founder event of HI population | Uniform | 400 - 3500 |
| Founder event of JP population | Uniform | 3000 - 80000 |
| Founder event of CN population | Uniform | 3000 - 80000 |
| Founder event of TH population | Uniform | 3000 - 80000 |
| Founder event of RE population | Uniform | 300 - 3000 |
| Founder event of AL population | Uniform | 40 - 400 |
| Founder event of GR population | Uniform | 10 - 150 |
| Rates of admixture, *ra* | Uniform | 0.001 - 0.999 |
| Mean mutation rate (MEANMU) | Uniform | 10^-5^ - 10^-3^ |
| Mean parameter of the geometric distribution of the number of repeats (MEAN P) | Uniform | 0.1 - 0.3 |
| Mean single nucleotide insertion/deletion mutation rate (MEANSNI) | Log-uniform | 10^-8^ - 10^-5^ |

*N_i_* = stable effective population size; *NF_i_* = effective numbers of founders during an introduction step lasting *Db_i_* generations, where *_I_*  = CN, JP, TH, RE, GR, AL, IT2, IT1, HI, VA; *ra* = admixture rate (only for scenarios with admixture); the times of introduction were translated into numbers of generations running back in time from the year of sampling (2010-2012) by assuming 4–7 generations per year in temperate regions, and 14–17 generations per year in tropical regions.
