# Supplementary material for: Genetic evidence for a worldwide chaotic dispersion pattern of the arbovirus vector, Aedes albopictus
Source: PLoS Negl Trop Dis. 2017 Jan 30;11(1):e0005332. doi: 10.1371/journal.pntd.0005332 (PMC5300280; doi:10.1371/journal.pntd.0005332)
Supplement: S2 Table — (DOCX) [file pntd.0005332.s004.docx]

**Table S2. Number of alleles per population at each microsatellite locus.**

|  | **JP** | **CN** | **TH** | **RE** | **GR** | **AL** | **IT1** | **IT2** | **HI** | **VA** |
| --- | --- | --- | --- | --- | --- | --- | --- | --- | --- | --- |
| **Aealbmic1** | 1 | 1 | 1 | 3 | 1 | 2 | 1 | 2 | 2 | 2 |
| **Aealbmic2** | 5 | 2 | 4 | 2 | 3 | 3 | 4 | 2 | 2 | 4 |
| **Aealbmic3** | 10 | 6 | 13 | 11 | 7 | 7 | 8 | 4 | 3 | 11 |
| **Aealbmic4** | 4 | 4 | 6 | 4 | 4 | 4 | 5 | 5 | 3 | 5 |
| **Aealbmic5** | 7 | 5 | 6 | 8 | 7 | 5 | 8 | 6 | 3 | 8 |
| **Aealbmic6** | 3 | 3 | 8 | 5 | 5 | 5 | 4 | 4 | 4 | 5 |
| **Aealbmic7** | 6 | 5 | 6 | 8 | 5 | 4 | 5 | 4 | 5 | 5 |
| **Aealbmic8** | 6 | 5 | 8 | 13 | 5 | 9 | 7 | 5 | 7 | 7 |
| **Aealbmic9** | 5 | 5 | 10 | 4 | 5 | 5 | 8 | 3 | 4 | 5 |
| **Aealbmic10** | 4 | 3 | 4 | 6 | 4 | 2 | 4 | 3 | 4 | 4 |
| **Aealbmic11** | 5 | 4 | 9 | 7 | 7 | 4 | 6 | 4 | 4 | 5 |
| **Aealbmic12** | 9 | 6 | 9 | 7 | 6 | 7 | 6 | 6 | 5 | 6 |
| **Aealbmic13** | 5 | 5 | 8 | 5 | 4 | 3 | 8 | 4 | 3 | 4 |
| **Aealbmic14** | 2 | 1 | 2 | 4 | 1 | 1 | 1 | 3 | 2 | 3 |
| **Aealbmic15** | 1 | 2 | 1 | 1 | 1 | 2 | 2 | 1 | 1 | 1 |
| **Aealbmic16** | 6 | 4 | 7 | 7 | 7 | 6 | 6 | 5 | 8 | 7 |
| **Aealbmic17** | 1 | 2 | 1 | 1 | 1 | 2 | 1 | 1 | 2 | 1 |

JP, Japan; CN, China; TH, Thailand; RE, La Réunion; GR, Greece; AL, Albania; IT1, Italy1/Cesena; IT2, Italy2/Brescia; HI, Hawaii; VA, Virginia (U.S.A.).
