# Supplementary material for: Genetic evidence for a worldwide chaotic dispersion pattern of the arbovirus vector, Aedes albopictus
Source: PLoS Negl Trop Dis. 2017 Jan 30;11(1):e0005332. doi: 10.1371/journal.pntd.0005332 (PMC5300280; doi:10.1371/journal.pntd.0005332)
Supplement: S3 Table — (DOC) [file pntd.0005332.s005.doc]

**S3 Table.** Average coefficient of ancestry obtained from a STRUCTURE run with *K* = 4 for 252 individuals of *Ae. albopictus* from 10 samples collected in different eco-geographical areas.

| Population | 1 | 2 | 3 | 4 |
| --- | --- | --- | --- | --- |
| JP | 0.077 | 0.524 | 0.087 | 0.312 |
| CN | 0.068 | 0.569 | 0.210 | 0.153 |
| TH | 0.340 | 0.303 | 0.164 | 0.194 |
| RE | 0.027 | 0.067 | 0.792 | 0.114 |
| GR | 0.827 | 0.054 | 0.025 | 0.094 |
| AL | 0.014 | 0.569 | 0.067 | 0.350 |
| IT1 | 0.018 | 0.381 | 0.286 | 0.315 |
| IT2 | 0.019 | 0.165 | 0.024 | 0.792 |
| HI | 0.029 | 0.049 | 0.025 | 0.898 |
| VA | 0.016 | 0.164 | 0.027 | 0.793 |
