# Supplementary material for: Genetic evidence for a worldwide chaotic dispersion pattern of the arbovirus vector, Aedes albopictus
Source: PLoS Negl Trop Dis. 2017 Jan 30;11(1):e0005332. doi: 10.1371/journal.pntd.0005332 (PMC5300280; doi:10.1371/journal.pntd.0005332)
Supplement: Supplementary file 5 [file pntd.0005332.s001.doc]

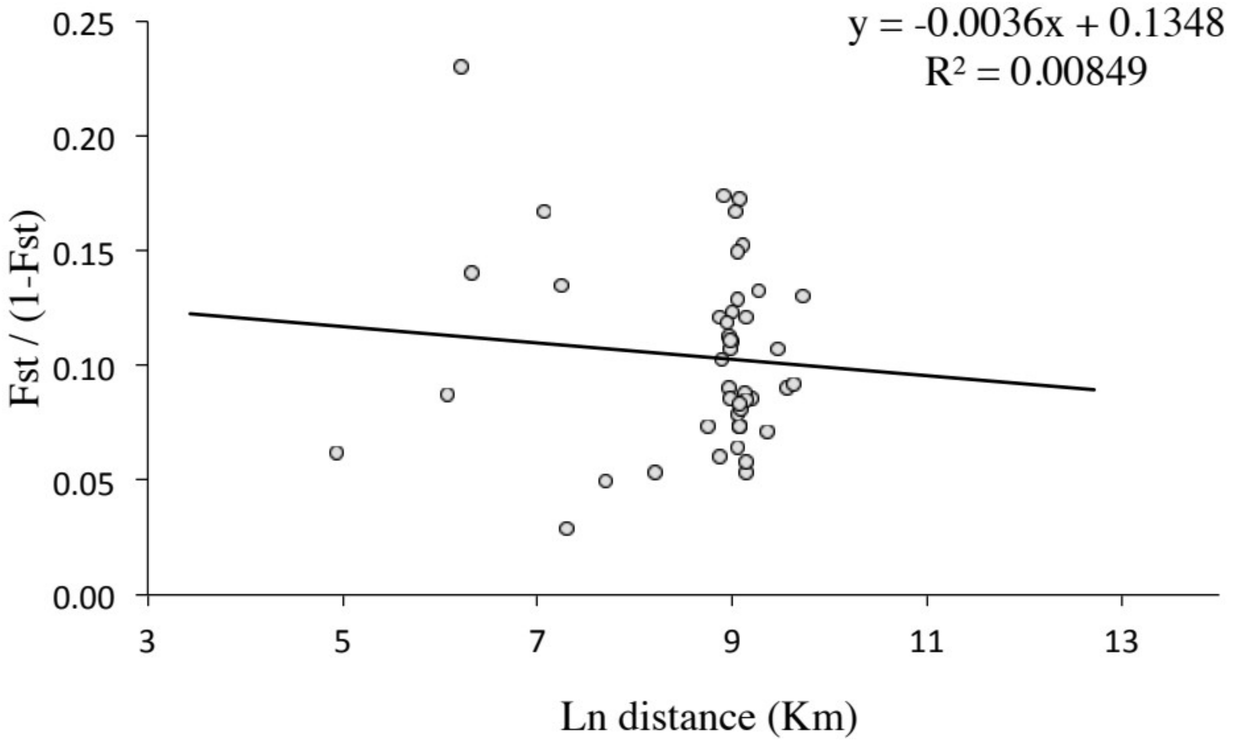
y = -0.0036x + 0.1348
R² = 0.00849
0.25
0.20
0.15
0.10
0.05
0.00
Fst / (1-Fst)
3
5
7
9
11
13
Ln distance (Km)
